# 2,2′-(Piperazine-1,4-di­yl)diethanaminium bis­(2-hy­droxy­benzoate)

**DOI:** 10.1107/S1600536812030103

**Published:** 2012-07-07

**Authors:** Ignacy Cukrowski, Adedapo S. Adeyinka, David C. Liles

**Affiliations:** a Department of Chemistry, University of Pretoria, Private Bag X20, Hatfield 0028, South Africa

## Abstract

The asymmetric unit of the title salt, C_8_H_22_N_4_^2+^·2C_7_H_5_O_3_^−^, comprises half a 2,2′-(piperazine-1,4-di­yl)diethan­aminium dication plus a 2-hy­droxy­benzoate anion. In the crystal, the anions and cations are linked by N—H⋯O and O—H⋯O hydrogen bonds to form infinite two-dimensional networks parallel with the *a* unit-cell face. The conformation adopted by the cation in the crystal is very similar to that adopted by the same cation in the structures of the nitrate and tetra­hydrogen penta­borate salts.

## Related literature

For the structures of the nitrate and tetra­hydrogen penta­borate salts of the 1,4-di(2-ammonio­eth­yl)piperazine cation, see: Junk & Smith (2005 ▶); Jiang *et al.* (2009 ▶); Yang *et al.* (2011 ▶).
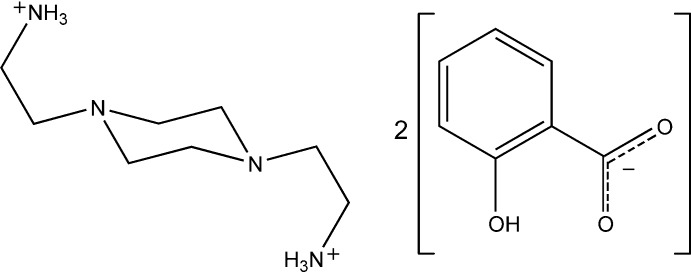


## Experimental

### 

#### Crystal data

C_8_H_22_N_4_^2+^·2C_7_H_5_O_3_^−^
*M* *_r_* = 448.52
Monoclinic, 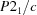

*a* = 11.5374 (4) Å
*b* = 6.4759 (2) Å
*c* = 15.5264 (6) Å
β = 104.207 (2)°
*V* = 1124.58 (7) Å^3^
*Z* = 2
Mo *K*α radiation
μ = 0.10 mm^−1^
*T* = 180 K
0.37 × 0.10 × 0.05 mm

#### Data collection

Nonius KappaCCD diffractometer
Absorption correction: multi-scan (*SORTAV*; Blessing, 1995 ▶) *T*_min_ = 0.852, *T*_max_ = 0.995
19655 measured reflections
3261 independent reflections
2013 reflections with *I* > 2σ(*I*)
*R*_int_ = 0.065

#### Refinement

*R*[*F* ^2^ > 2σ(*F* ^2^)] = 0.050
*wR*(*F* ^2^) = 0.147
*S* = 1.08
3261 reflections
157 parameters
H atoms treated by a mixture of independent and constrained refinement
Δρ_max_ = 0.34 e Å^−3^
Δρ_min_ = −0.28 e Å^−3^

### 

Data collection: *COLLECT* (Nonius, 1998 ▶); cell refinement: *SCALEPACK* (Otwinowski & Minor, 1997 ▶); data reduction: *DENZO* (Otwinowski & Minor, 1997 ▶), *SCALEPACK* and *SORTAV* (Blessing, 1995 ▶); program(s) used to solve structure: *SIR92* (Altomare *et al.*, 1994 ▶); program(s) used to refine structure: *SHELXL97* (Sheldrick, 2008 ▶); molecular graphics: *ORTEP-3 for Windows* (Farrugia, 1997 ▶), *POV-RAY* (Cason, 2004 ▶) and *Mercury* (Macrae *et al.*, 2008 ▶); software used to prepare material for publication: *SHELXL97* and *PLATON* (Spek, 2009 ▶).

## Supplementary Material

Crystal structure: contains datablock(s) I, global. DOI: 10.1107/S1600536812030103/jj2133sup1.cif

Structure factors: contains datablock(s) I. DOI: 10.1107/S1600536812030103/jj2133Isup2.hkl

Supplementary material file. DOI: 10.1107/S1600536812030103/jj2133Isup3.cml

Additional supplementary materials:  crystallographic information; 3D view; checkCIF report

## Figures and Tables

**Table 1:** Hydrogen-bond geometry (Å, °)

*D*—H⋯*A*	*D*—H	H⋯*A*	*D*⋯*A*	*D*—H⋯*A*
N1—H1*A*⋯O14	0.929 (17)	1.992 (18)	2.8853 (17)	160.9 (14)
N1—H1*B*⋯O14^i^	0.900 (18)	1.923 (19)	2.7909 (17)	161.5 (14)
N1—H1*C*⋯O15^ii^	0.892 (18)	1.902 (19)	2.7843 (17)	169.9 (15)
O16—H16⋯O15	0.81 (2)	1.83 (2)	2.5641 (16)	149.4 (19)

Symmetry codes: (i) 
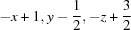
; (ii) 
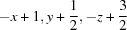
.

**Fig. 1.**
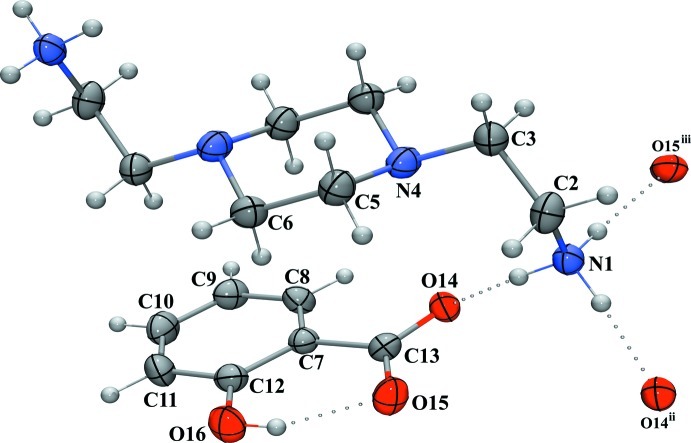
Molecular structure of the title compound showing the atom labeling scheme and 50° probability displacement ellipsoids. Broken lines indicate N—H···O and O—H···O hydrogen bonds. Symmetry codes: (ii) -x + 1, y - 1/2, -z + 3/2; (iii) -x + 1, y + 1/2, -z + 3/2.

**Fig. 2.**
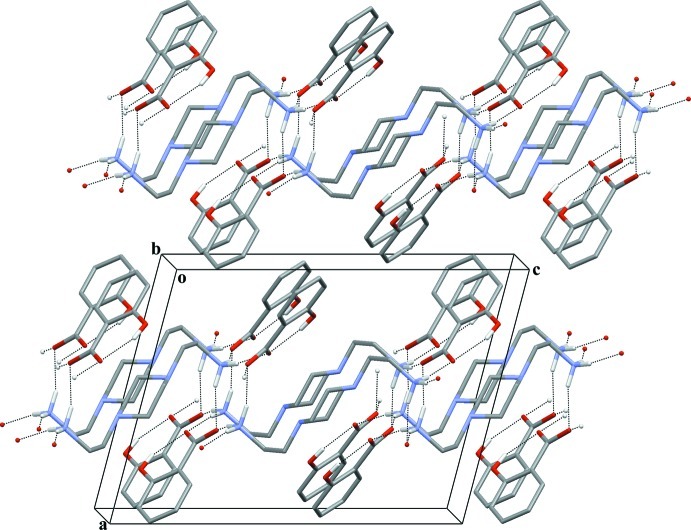
Packing diagram of the title compound viewed offset from along the b axis. Dashed lines indicate N—H···O and O—H···O hydrogen bonds. The intermolecular N—H···O hydrogen bonds form a two-dimensional network.

**Fig. 3.**
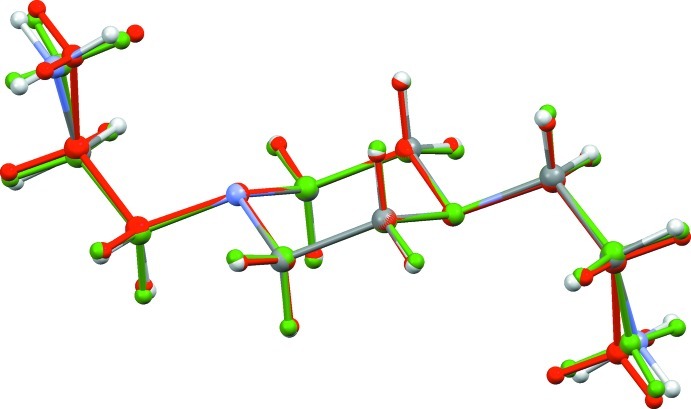
The structures of the 1,4-di(2-ammonioethyl)piperazine cations of the C7H5O3- (standard atom colours, 1), the NO3- (green, Junk & Smith, 2005) and the H4B5O10- (red, Jiang, et al., 2009) salts, superimposed on each other to show the similar conformations adopted by the cations in all three structures.

## supplementary crystallographic information

### Comment

The title compound [C_8_H_22_N_4_^2+^ 2(C_7_H_5_O_3_^-^)] (1) was obtained as an unintended product during an attempt to prepare a 2-hydroxybenzoate salt of a singly protonated *N*,*N*'-di(2-aminoethyl)-2-aminoethane-1-ammonium ion (C_6_H_19_N_4_^+^ C_7_H_5_O_3_^-^). This occurred because the starting material, instead of being pure *N*,*N*'-di(2-aminoethyl)-ethane-1,2-diamine (C_6_H_18_N_4_), was a mixture of that compound and 1,4-di(2-aminoethyl)piperazine (C_8_H_22_N_4_). A similar situation appears to have occurred for a published structure which the authors (Yang, *et al.*, 2011) claim to be a *N*,*N*'-di(2-ammonioethyl)-ethane-1,2-diamine (*i.e*. a doubly protonated ion derived from C_6_H_18_N_4_) salt of tetrahydrogenpentaborate (H_4_B_5_O_10_^-^) but with the central C_2_H_4_ moiety disordered over two sites. In fact the two "disordered" C_2_H_4_ sites together with the two adjacent N atoms form the central piperazine ring of a 1,4-di(2-ammonioethyl)piperazine ion (C_8_H_22_N_4_^2+^) and the reported crystal structure is identical (within experimental error) with that of C_8_H_22_N_4_^2+^ 2(H_4_B_5_O_10_^-^) (Jiang, *et al.*, 2009).

The C_8_H_22_N_4_^2+^ cation in 1 is symmetrical and lies across a crystallographic centre of inversion. Each ammonium group in the cations of 1 is the donor for three hydrogen bonds to the O atoms of the carboxylate groups of three different 2-hydroxybenzoate anions (Fig. 1). There is also an intra-molecular hydrogen bond between the hydroxy group and one of the carboxylate O atoms in the C_7_H_5_O_3_^-^ anions. Thus both the O atoms of each carboxylate group are each acceptors for two hydrogen bonds. The hydrogen bonds link the cations and anions to form two-dimensional networks with the layers parallel with the *A* face of the unit cell (Fig. 2).

Fig. 3 illustrates that the conformation adopted by the C_8_H_22_N_4_^2+^ cation in the crystal structure of 1 is very similar to the conformations adopted by the same cation in the crystal structures of the NO_3_^-^ (Junk & Smith, 2005) and the H_4_B_5_O_10_^-^ (Jiang, *et al.*, 2009) salts despite the differences in the size and shape of the anions in the various structures.

### Experimental

2 ml of a 3.32 *M* aqueous solution of what was claimed by the supplier (QinHuangDao JinLei Chemical Co.Ltd) to be *N*,*N*'-di(2-aminoethyl)-ethane-1,2-diamine, but which turned out to be a mixture of that compound (C_6_H_18_N_4_, 6.64*n* mmol) and 1,4-di(2-aminoethyl)piperazine (C_8_H_20_N_4_, 5.57(1-*n*) mmol) was added to 0.96 g of 2-hydroxybenzoic acid (6.95 mmol), resulting in a clear colourless solution. 0.2 ml of ethanol was added to the solution and the mixture was heated for 3 h at 70 °C, the solution turned greenish yellow after one hour of heating. It was cooled to room temperature and then left covered for six days and then allowed to slowly evaporate by covering the container with perforated aluminium foil. Yellow crystals were obtained after four days of slow evaporation.

### Refinement

H1A, H1B and H1C were located by a difference map and their coordinates were refined. All of the remaining H atoms were placed in their calculated positions and then refined using the riding model with Atom—H lengths of 0.95 Å, (CH) or 0.99 Å (CH_2_). Isotropic displacement parameters for all hydrogen atoms were set to 1.20 times *U*_eq_ of the parent atom.

### Crystal data

**Table:**

C_8_H_22_N_4_^2+^·2C_7_H_5_O_3_^−^	*F*(000) = 480
*M**_r_* = 448.52	*D*_x_ = 1.325 Mg m^−^^3^
Monoclinic, *P*2_1_/*c*	Mo *K*α radiation, λ = 0.71073 Å
Hall symbol: -P 2ybc	Cell parameters from 26933 reflections
*a* = 11.5374 (4) Å	θ = 1.0–30.0°
*b* = 6.4759 (2) Å	µ = 0.10 mm^−^^1^
*c* = 15.5264 (6) Å	*T* = 180 K
β = 104.207 (2)°	Block, yellow
*V* = 1124.58 (7) Å^3^	0.37 × 0.10 × 0.05 mm
*Z* = 2	

### Data collection

**Table:**

Nonius KappaCCD diffractometer	3261 independent reflections
Radiation source: fine-focus sealed tube	2013 reflections with *I* > 2σ(*I*)
Graphite monochromator	*R*_int_ = 0.065
Thin slice ω and φ scans	θ_max_ = 30.0°, θ_min_ = 3.6°
Absorption correction: multi-scan (*SORTAV*; Blessing, 1995)	*h* = −16→16
*T*_min_ = 0.852, *T*_max_ = 0.995	*k* = −8→9
19655 measured reflections	*l* = −21→21

### Refinement

**Table:**

Refinement on *F*^2^	Primary atom site location: structure-invariant direct methods
Least-squares matrix: full	Secondary atom site location: difference Fourier map
*R*[*F*^2^ > 2σ(*F*^2^)] = 0.050	Hydrogen site location: difference Fourier map
*wR*(*F*^2^) = 0.147	H atoms treated by a mixture of independent and constrained refinement
*S* = 1.08	*w* = 1/[σ^2^(*F*_o_^2^) + (0.0714*P*)^2^ + 0.0149*P*] where *P* = (*F*_o_^2^ + 2*F*_c_^2^)/3
3261 reflections	(Δ/σ)_max_ < 0.001
157 parameters	Δρ_max_ = 0.34 e Å^−^^3^
0 restraints	Δρ_min_ = −0.28 e Å^−^^3^

### Special details

**Table:**

Experimental. The –OH and –NH_3_ hydrogen atoms were located and their positions were refined satisfactorily.
Geometry. All e.s.d.'s (except the e.s.d. in the dihedral angle between two l.s. planes) are estimated using the full covariance matrix. The cell e.s.d.'s are taken into account individually in the estimation of e.s.d.'s in distances, angles and torsion angles; correlations between e.s.d.'s in cell parameters are only used when they are defined by crystal symmetry. An approximate (isotropic) treatment of cell e.s.d.'s is used for estimating e.s.d.'s involving l.s. planes.
Refinement. Refinement of *F*^2^ against ALL reflections. The weighted *R*-factor *wR* and goodness of fit *S* are based on *F*^2^, conventional *R*-factors *R* are based on *F*, with *F* set to zero for negative *F*^2^. The threshold expression of *F*^2^ > σ(*F*^2^) is used only for calculating *R*-factors(gt) *etc*. and is not relevant to the choice of reflections for refinement. *R*-factors based on *F*^2^ are statistically about twice as large as those based on *F*, and *R*- factors based on ALL data will be even larger.

### Fractional atomic coordinates and isotropic or equivalent isotropic displacement parameters (Å^2^)

**Table:**

	*x*	*y*	*z*	*U*_iso_*/*U*_eq_	
N1	0.39360 (12)	0.1995 (2)	0.71495 (9)	0.0296 (3)	
H1A	0.4724 (16)	0.215 (2)	0.7117 (11)	0.036*	
H1B	0.3890 (14)	0.076 (3)	0.7403 (10)	0.036*	
H1C	0.3822 (14)	0.299 (3)	0.7516 (11)	0.036*	
C2	0.30579 (14)	0.2076 (2)	0.62703 (10)	0.0355 (4)	
H2A	0.2242	0.1866	0.6352	0.043*	
H2B	0.3228	0.0945	0.5890	0.043*	
C3	0.31138 (13)	0.4123 (2)	0.58149 (11)	0.0328 (4)	
H3A	0.2467	0.4186	0.5262	0.039*	
H3B	0.2985	0.5257	0.6209	0.039*	
N4	0.42749 (11)	0.44057 (19)	0.55982 (8)	0.0321 (3)	
C5	0.43393 (14)	0.3166 (2)	0.48157 (11)	0.0350 (4)	
H5A	0.3691	0.3593	0.4302	0.042*	
H5B	0.4219	0.1691	0.4937	0.042*	
C6	0.44661 (14)	0.6567 (2)	0.54111 (10)	0.0332 (4)	
H6A	0.4446	0.7419	0.5937	0.040*	
H6B	0.3817	0.7044	0.4907	0.040*	
C7	0.80219 (12)	0.2749 (2)	0.66915 (9)	0.0261 (3)	
C8	0.84808 (13)	0.4670 (2)	0.70044 (10)	0.0307 (3)	
H8	0.8129	0.5394	0.7408	0.037*	
C9	0.94297 (13)	0.5555 (2)	0.67466 (11)	0.0356 (4)	
H9	0.9731	0.6863	0.6973	0.043*	
C10	0.99343 (14)	0.4510 (3)	0.61536 (11)	0.0390 (4)	
H10	1.0584	0.5111	0.5968	0.047*	
C11	0.95065 (15)	0.2608 (3)	0.58287 (11)	0.0381 (4)	
H11	0.9861	0.1908	0.5420	0.046*	
C12	0.85550 (13)	0.1702 (2)	0.60957 (10)	0.0306 (3)	
C13	0.69936 (13)	0.1830 (2)	0.69894 (9)	0.0273 (3)	
O14	0.64524 (9)	0.28943 (15)	0.74448 (7)	0.0321 (3)	
O15	0.67079 (11)	−0.00229 (16)	0.67621 (7)	0.0393 (3)	
O16	0.81677 (11)	−0.01682 (18)	0.57563 (8)	0.0418 (3)	
H16	0.7652 (17)	−0.055 (3)	0.5996 (14)	0.050*	

### Atomic displacement parameters (Å^2^)

**Table:**

	*U*^11^	*U*^22^	*U*^33^	*U*^12^	*U*^13^	*U*^23^
N1	0.0299 (7)	0.0264 (7)	0.0360 (7)	−0.0006 (6)	0.0145 (6)	0.0013 (6)
C2	0.0369 (8)	0.0391 (9)	0.0326 (8)	−0.0098 (7)	0.0124 (7)	−0.0004 (7)
C3	0.0312 (8)	0.0365 (9)	0.0327 (8)	0.0006 (7)	0.0115 (6)	0.0030 (7)
N4	0.0301 (7)	0.0310 (7)	0.0359 (7)	−0.0020 (5)	0.0091 (5)	0.0064 (6)
C5	0.0381 (8)	0.0336 (8)	0.0345 (9)	−0.0029 (7)	0.0111 (7)	0.0020 (7)
C6	0.0362 (8)	0.0299 (8)	0.0339 (8)	0.0013 (7)	0.0094 (7)	0.0017 (7)
C7	0.0249 (7)	0.0260 (7)	0.0270 (7)	0.0011 (6)	0.0057 (6)	0.0028 (6)
C8	0.0265 (7)	0.0302 (8)	0.0359 (8)	0.0007 (6)	0.0084 (6)	−0.0030 (7)
C9	0.0298 (8)	0.0322 (8)	0.0435 (9)	−0.0052 (7)	0.0068 (7)	0.0011 (7)
C10	0.0289 (8)	0.0464 (10)	0.0434 (9)	−0.0028 (7)	0.0123 (7)	0.0066 (8)
C11	0.0355 (8)	0.0446 (10)	0.0387 (9)	0.0066 (7)	0.0180 (7)	0.0010 (7)
C12	0.0332 (8)	0.0278 (7)	0.0309 (8)	0.0021 (6)	0.0076 (6)	0.0013 (6)
C13	0.0294 (7)	0.0254 (7)	0.0266 (7)	−0.0011 (6)	0.0061 (6)	0.0025 (6)
O14	0.0321 (6)	0.0299 (6)	0.0379 (6)	0.0002 (4)	0.0154 (5)	−0.0010 (5)
O15	0.0528 (7)	0.0291 (6)	0.0409 (6)	−0.0125 (5)	0.0210 (5)	−0.0062 (5)
O16	0.0532 (8)	0.0314 (6)	0.0466 (7)	−0.0046 (5)	0.0234 (6)	−0.0111 (5)

### Geometric parameters (Å, º)

**Table:**

N1—C2	1.488 (2)	C6—H6B	0.9900
N1—H1A	0.929 (17)	C7—C8	1.392 (2)
N1—H1B	0.900 (18)	C7—C12	1.404 (2)
N1—H1C	0.892 (18)	C7—C13	1.498 (2)
C2—C3	1.511 (2)	C8—C9	1.379 (2)
C2—H2A	0.9900	C8—H8	0.9500
C2—H2B	0.9900	C9—C10	1.381 (2)
C3—N4	1.4706 (19)	C9—H9	0.9500
C3—H3A	0.9900	C10—C11	1.376 (2)
C3—H3B	0.9900	C10—H10	0.9500
N4—C6	1.4572 (19)	C11—C12	1.394 (2)
N4—C5	1.473 (2)	C11—H11	0.9500
C5—C6^i^	1.514 (2)	C12—O16	1.3524 (18)
C5—H5A	0.9900	C13—O14	1.2575 (17)
C5—H5B	0.9900	C13—O15	1.2714 (17)
C6—C5^i^	1.514 (2)	O16—H16	0.81 (2)
C6—H6A	0.9900		
			
C2—N1—H1A	113.7 (10)	N4—C6—H6A	109.6
C2—N1—H1B	109.4 (10)	C5^i^—C6—H6A	109.6
H1A—N1—H1B	106.6 (14)	N4—C6—H6B	109.6
C2—N1—H1C	112.7 (10)	C5^i^—C6—H6B	109.6
H1A—N1—H1C	104.7 (15)	H6A—C6—H6B	108.1
H1B—N1—H1C	109.4 (15)	C8—C7—C12	118.06 (14)
N1—C2—C3	111.24 (13)	C8—C7—C13	120.61 (13)
N1—C2—H2A	109.4	C12—C7—C13	121.33 (13)
C3—C2—H2A	109.4	C9—C8—C7	122.09 (14)
N1—C2—H2B	109.4	C9—C8—H8	119.0
C3—C2—H2B	109.4	C7—C8—H8	119.0
H2A—C2—H2B	108.0	C8—C9—C10	118.95 (15)
N4—C3—C2	111.24 (12)	C8—C9—H9	120.5
N4—C3—H3A	109.4	C10—C9—H9	120.5
C2—C3—H3A	109.4	C11—C10—C9	120.74 (15)
N4—C3—H3B	109.4	C11—C10—H10	119.6
C2—C3—H3B	109.4	C9—C10—H10	119.6
H3A—C3—H3B	108.0	C10—C11—C12	120.36 (15)
C6—N4—C3	110.87 (12)	C10—C11—H11	119.8
C6—N4—C5	108.55 (12)	C12—C11—H11	119.8
C3—N4—C5	110.73 (11)	O16—C12—C11	118.04 (14)
N4—C5—C6^i^	111.26 (12)	O16—C12—C7	122.17 (13)
N4—C5—H5A	109.4	C11—C12—C7	119.79 (14)
C6^i^—C5—H5A	109.4	O14—C13—O15	122.86 (13)
N4—C5—H5B	109.4	O14—C13—C7	119.76 (13)
C6^i^—C5—H5B	109.4	O15—C13—C7	117.39 (13)
H5A—C5—H5B	108.0	C12—O16—H16	107.4 (14)
N4—C6—C5^i^	110.31 (13)		
			
N1—C2—C3—N4	64.77 (17)	C9—C10—C11—C12	−0.1 (2)
C2—C3—N4—C6	−162.96 (13)	C10—C11—C12—O16	179.96 (14)
C2—C3—N4—C5	76.48 (16)	C10—C11—C12—C7	0.7 (2)
C6—N4—C5—C6^i^	58.44 (17)	C8—C7—C12—O16	−179.88 (13)
C3—N4—C5—C6^i^	−179.64 (12)	C13—C7—C12—O16	0.5 (2)
C3—N4—C6—C5^i^	−179.69 (12)	C8—C7—C12—C11	−0.7 (2)
C5—N4—C6—C5^i^	−57.85 (17)	C13—C7—C12—C11	179.64 (13)
C12—C7—C8—C9	0.1 (2)	C8—C7—C13—O14	8.1 (2)
C13—C7—C8—C9	179.72 (13)	C12—C7—C13—O14	−172.27 (13)
C7—C8—C9—C10	0.5 (2)	C8—C7—C13—O15	−172.00 (13)
C8—C9—C10—C11	−0.5 (2)	C12—C7—C13—O15	7.6 (2)

Symmetry code: (i) −*x*+1, −*y*+1, −*z*+1.

### Hydrogen-bond geometry (Å, º)

**Table:**

*D*—H···*A*	*D*—H	H···*A*	*D*···*A*	*D*—H···*A*
N1—H1*A*···O14	0.929 (17)	1.992 (18)	2.8853 (17)	160.9 (14)
N1—H1*B*···O14^ii^	0.900 (18)	1.923 (19)	2.7909 (17)	161.5 (14)
N1—H1*C*···O15^iii^	0.892 (18)	1.902 (19)	2.7843 (17)	169.9 (15)
O16—H16···O15	0.81 (2)	1.83 (2)	2.5641 (16)	149.4 (19)

Symmetry codes: (ii) −*x*+1, *y*−1/2, −*z*+3/2; (iii) −*x*+1, *y*+1/2, −*z*+3/2.

## References

[bb1] Altomare, A., Cascarano, G., Giacovazzo, C., Guagliardi, A., Burla, M. C., Polidori, G. & Camalli, M. (1994). *J. Appl. Cryst.* **27**, 435.

[bb2] Blessing, R. H. (1995). *Acta Cryst.* A**51**, 33–38. 10.1107/s0108767394005726 7702794

[bb3] Cason, C. J. (2004). *POV-RAY for Windows* Persistence of Vision Raytracer Pty Ltd, Victoria, Australia. URL: http://www.povray.org.

[bb4] Farrugia, L. J. (1997). *J. Appl. Cryst.* **30**, 565.

[bb5] Jiang, X., Liu, H.-X., Wu, S.-L. & Liang, Y.-X. (2009). *Jiegou Huaxue* (*Chin. J. Struct. Chem*), **28**, 723–729.

[bb6] Junk, P. C. & Smith, M. K. (2005). *C. R. Chim.* **8**, 189–198.

[bb7] Macrae, C. F., Bruno, I. J., Chisholm, J. A., Edgington, P. R., McCabe, P., Pidcock, E., Rodriguez-Monge, L., Taylor, R., van de Streek, J. & Wood, P. A. (2008). *J. Appl. Cryst.* **41**, 466–470.

[bb8] Nonius (1998). *COLLECT* Nonius BV, Delft, The Netherlands.

[bb9] Otwinowski, Z. & Minor, W. (1997). *Methods in Enzymology*, Vol. 276, *Macromolecular Crystallography*, Part A, edited by C. W. Carter Jr & R. M. Sweet, pp. 307–326. New York: Academic Press.

[bb10] Sheldrick, G. M. (2008). *Acta Cryst.* A**64**, 112–122. 10.1107/S0108767307043930 18156677

[bb11] Spek, A. L. (2009). *Acta Cryst.* D**65**, 148–155. 10.1107/S090744490804362X PMC2631630 19171970

[bb12] Yang, Y., Sun, J.-B., Cui, M., Liu, R.-B., Wang, Y. & Meng, C.-G. (2011). *J. Solid State Chem.* **184**, 1666–1670.

